# NONHSAT021545/miR-330-3p/EREG: A Cooperative Axis in Breast Cancer Prognosis and Treatment

**DOI:** 10.3390/jcm12072478

**Published:** 2023-03-24

**Authors:** Yunkun Zhang, Chunmei Guo, Siwen Yang, Maroua Elkharti, Rui Liu, Ming-Zhong Sun, Shuqing Liu

**Affiliations:** 1 Department of Biochemistry, College of Basic Medical Sciences, Dalian Medical University, Dalian 116044, China; 2 Department of Pathology, The Second Affiliated Hospital of Dalian Medical University, Dalian 116023, China; 3 Department of Biotechnology, College of Basic Medical Sciences, Dalian Medical University, Dalian 116044, China

**Keywords:** breast cancer, miR-330-3p, lnc021545, EREG, coordinated function of multi-genes

## Abstract

Lymphatic metastasis is the most common form in breast cancer (BC) progression. Previously, we observed that lnc045874, a most conservative homology of Homo Sapiens NONHSAT021545 (lnc021545), miR-330-3p, and EREG may have some effects in mouse hepatocarcinoma cell lines with different lymphatic metastasis potentials. Through data from TCGA and GEO database analysis, we speculated that miR-330-3p might be a tumor promoter, while EREG could be a tumor suppressor in BC. MiR-330-3p was upregulated, while lnc021545 and EREG were downregulated in 50 BC tissues. MiR-330-3p advanced the metastatic behaviors of BC cells, whereas lnc021545 and EREG resulted in the opposite effects. The three molecules’ expressions were correlated respectively and showed that miR-330-3p targeted lnc021545 and EREG to affect their expressions. Lnc021545/miR-330-3p axis affected BC metastasis by regulating EREG in epithelial-to-mesenchymal transition. In 50 BC patients, these three molecules and their cooperation are associated with aggressive tumor phenotypes, patient outcomes, and trastuzumab therapy. We finally discovered that lnc021545, miR-330-3p, and EREG formed a multi-gene co-regulation system that affected the metastasis of BC and the cooperation reflects the synergistic effects of the three molecules, recommending that their cooperation may provide a more accurate index for anti-metastasis therapeutic and prognostic evaluation of BC.

## 1. Introduction

In female malignancies, breast cancer (BC) is considered to be the most frequently diagnosed cancer [1]. For cancer patients, distal metastases and resistance to therapeutics are the leading causes of death [2]. Biologically, distant cancer metastasis involves a variety of complex mechanisms, such as EMT, migration, invasion, angiogenesis, and adhesion [3,4]. Interpreting these mechanisms can be beneficial for discovering new therapeutic regimens and markers.

Lymphatic metastasis is the most common form of BC progression [5]. In our previous research, the differentially expressed miRNAs, lncRNAs, and mRNAs in mouse hepatocarcinoma Hca-F (with high lymphatic metastasis potential) and Hca-P (with low lymphatic metastasis potential) cell lines were screened using a high-throughput RNA sequencing method. Among them, miR-330-3p, lnc045874, and EREG have shown significantly different expressions between the two cell lines. Murine lnc045874 is the most conservative homology of human NONHSAT021545 (lnc021545). Meanwhile, lnc021545 conserved same the binding site as miR-330-3p. TargetScan (http://www.targetscan.org accessed on 1 March 2020) analysis suggested that miR-330-3p has two combining sites with the 3′-untranslated region (3′-UTR) of EREG and one combining site with lnc045874. We aimed to determine whether these three molecules could synergistically affect the progression of BC by modulating the lymphatic metastasis and evaluated the clinical feature of BC patients.

The microRNAs (miRNAs) are a type of small, non-coding RNA composed of 18–24 nucleotides. The miRNAs can negatively modulate genes’ expressions by binding their 3′-UTRs, which act as crucial effectors in the malignant behaviors of tumor cells [6]. MiR-330-3p is located on chromosome 19q12.32 [7] and is bound to the malignant behaviors of cancerous cells; It plays a moderator role in different cancers [8]. Long non-coding RNAs (lncRNAs) are a type of RNA transcript with a length longer than 200 nucleotides without protein-coding ability [9]. LncRNAs have been demonstrated to modulate the biological functions of mRNAs by combing miRNAs [10]. Lnc021545 (NONCODE ID: NONHSAT021545.2) is located on human chromosome 11 with a length of 3149 bases; the exact role of lnc021545 in BC remains elusive. Epiregulin (EREG) is located on chromosome 4q13.3 and is a member of the epidermal growth factor family [11]. EREG acts as a crucial effector in the modulation of angiogenesis [12], inflammation [13], drug resistance [14], and metastasis [15], whereas the effect of EREG in the origination and progression of BC remains unconfident.

The development of a disease is a complex process caused by the dysregulation of multiple genes’ functions rather than by the dysregulation of a single or an isolated gene’s functions [16]. With the identification of a mass of cancer-related genes and their functions, the studies that coordinated function of multi-genes influence the prognosis and treatment of cancer are increasingly emerging. In the research of BC, the expression levels of 21 genes associated with the biological characteristics of BC are detected via RT-PCR, and the recurrence score (RS) is obtained using an algorithm based on previous clinical studies [17]. The RS has been integrated into the National Comprehensive Cancer Network clinical guidelines for treatment and predictive prognosis of hormone-receptor-positive and HER-2-negative diseases [18]. By studying the effects of lnc021545, miR-330-3p, and EREG in BC, we will explore whether these three molecules’ coordinated functions could affect BC’s prognosis and treatment.

As part of this study, we established a new lnc021545-miR-330-3p-EREG axis regulatory network in BC. We found that miR-330-3p was upregulated, while lnc021545 and EREG were downregulated in BC patients’ samples. Moreover, lnc021545 and EREG act as tumor suppressors in regulating the metastasis of BC cells, while miR-330-3p acts as a tumor promoter and showed opposite effects on BC cells. Furthermore, lnc021545/miR-330-3p affects the EMT process of BC cells by regulating EREG expression. Our study demonstrates lnc021545-miR-330-3p-EREG axis regulatory network in BC progression and provides a new clue to the treatment and prognosis of BC.

## 2. Materials and Methods

### 2.1. Tissue Samples and Cell Culture

Fifty pairs of BC tissues with their one-to-one corresponding adjacent non-tumor tissues were collected from patients who underwent surgical resection at the Second Affiliated Hospital of Dalian Medical University between 2015 and 2019. The study was approved by the Medical Ethics Committee of Dalian Medical University (approval number 2019-069). The informed consent forms were signed by all participants. After resection, the tissue specimens were stored at −80 °C until use. Human BC cell lines MCF-7 and T47D were obtained from the American Type Culture Collection. Both cell lines were cultured in RPMI 1640 (Gibco, New York, NY, USA) supplemented with 15% fetal bovine serum (FBS, TransGen, Beijing, China) and 100 U/mL penicillin/streptomycin (Gibco) at 37 °C with 5% CO_2_.

### 2.2. Bioinformatic Analysis

The data of miRNA-seq were downloaded from The Cancer Genome Atlas (TCGA, https://tcgadata.nci.nih.gov/tcga accessed on 18 September 2022). We also queried the Gene Expression Omnibus (GEO, https://www.ncbi.nlh.gov/geo accessed on 20 September 2022) database for miRNA and mRNA profiling by arrays. We analyzed the expression data of 1103 BC tissues and 104 non-tumor tissues from the TCGA-BRCA dataset by employing R package “edgeR” to identify and normalize the differentially expressed miRNAs. GSE22216 and GSE40267 were utilized for survival analysis according to miR-330-3p expression changes. To evaluate the prognostic value of EREG, survival analysis was performed using the web tool KMplot (http://kmplot.com accessed on 26 September 2022). Gene set enrichment analysis (GSEA) was executed on the GSE3494 dataset to analyze the associated functional gene sets in BC pathogenesis.

### 2.3. Transient Transfection

Two small interfering RNAs of lnc021545 (si-lnc021545), three small interfering RNAs of EREG (si-EREG), siRNA negative control (si-NC), mimic or inhibitor of miR-330-3p, miRNA negative control (NC), PCDH-EF1-MCS-T2A-Puro-EREG (PCDH-EREG), and PCDH-EF1-MCS-T2A-Puro vector (PCDH) were used, and the sequences of si-RNAs for EREG and lnc021545 knockdown are shown in Table S1.

In total, 3 × 10^5^ MCF-7 and T47D cells were seeded per well and incubated at 37 °C with 5% CO_2_ for 12 h. To downregulate lnc021545 in cells, 3.5 µL si-lnc021545-1 and 3.5 µL si-lnc021545-2 or 7 µL si-NC with a consistency of 20 µM and the supplementary of 6 µL Lipofectamine™ 2000 (Lipo2000, Invitrogen, Waltham, MA, USA) were mixed to form a transfection mixture and kept still for 20 min at room temperature (RT). Then, 5 µL miR-330-3p mimic, mimic NC, miR-330-3p inhibitor or inhibitor NC with a consistency of 20 µM, and the supplementary of 5 µL Lipo2000 were mixed to form a transfection mixture to overexpress and downregulate miR-330-3p in cells. An amount of 1 µg PCDH-EREG or 1 µg PCDH was mixed with 50 µL serum-free medium, 5 µL Lipo2000 was mixed with 45 µL serum-free medium, and the above two mixtures were mixed in order to establish the cells of EREG over-expression and placed for 20 min at RT. 2.5 µL si-EREG-1, si-EREG-2, and si-EREG-3 or 7.5 µL si-NC with a consistency of 20 µM. A supplement of 6 µL Lipo2000 was mixed and placed for 20 min at RT as the transfection mixture to EREG downregulation. Each of the transfection mixtures were added into each group of MCF-7 or T47D cells. Then, the cells were cultured at 37 °C under 5% CO_2_ for 24 h or 48 h for further experiments.

### 2.4. Cell Proliferation Assay

The proliferation abilities of different group of MCF-7 and T47D cells were measured via MTT assay. Each group was seeded with a density of 3 × 10^3^ cells/well in a 96-well plate (NEST, Wuxi, China). MTT reagent was added to each well in 200 μL increments at 24 h, 48 h, 72 h, 96 h, and 120 h. After 4 h, the MTT reagent was substituted with 150 μL dimethyl sulfoxide (Sigma, St. Louis, MO, USA). The absorbance of 492 nm was detected using a plate reader (Eppendorf, Hamburg, Germany) for the quantification of cell viability.

### 2.5. Cell Motility Assay

The motility ability of MCF-7 cells was measured by wound-healing experiments. In total, 6 × 10^5^ of each group of MCF-7 cells were seeded into a 6-well plate (NEST) at 37 °C with 5% CO_2_ and cultured to 85–90% confluence. Then, a 200 μL sterilized tip drew vertical scratches on the monolayer cell surface. Detached and floated cells were washed with PBS, then cultured at 37 °C for 24 h. The widths of scratch wounds were obtained under a microscope (Olympus, Tokyo, Japan) at 100× magnification at 0 h, 24 h, and assessed using Image J software (version 2.3.0).

### 2.6. Cell Migration and Invasion Assay

The migration and invasion of cells were tested via transwell chamber assay. For migration assay, 1 × 10^4^ transfected MCF-7 or T47D cells in 200 µL RPMI 1640 with serum-free media were seeded in the upper chamber of the transwell. For invasion assay, the chambers were layered in an extracellular matrix (Sigma). The upper chamber was assembled into the plate wells, and the bottom chamber was filled with 600 μL RPMI 1640 with 20% FBS and cultured for 48 h at 37 °C with 5% CO_2_. The migrative or invasive cells through the membrane were fixed for 30 min in absolute methanol and stained in 0.5% crystal violet stain solution (Solarbio, Beijing, China) for 1 h at RT, then imaged and counted with the microscope (Olympus) at 100× magnification.

### 2.7. Western Blotting (WB) Assay

RIPA lysis buffer was used to extract the total proteins of transfected BC cells. The quantitation of protein was evaluated using Bradford assay [19]. After, the sample of protein (30 µg) was separated using 10% sodium dodecyl sulphate-polyacrylamide gels and transferred onto nitrocellulose membrane (PALL, Port Washington, NY, USA), followed by a blocking at RT with 5% skim milk for 2 h. Then, the membranes were incubated with the following primary antibodies: EREG (Abcam, Cambridge, MA, USA), E-cadherin (ProteinTech, Wuhan, China), N-cadherin (ProteinTech), vimentin (ProteinTech), Snail (ProteinTech), Slug (ProteinTech), and GAPDH (ProteinTech) overnight at 4 °C. After washing three times with TBST, the membranes were incubated in the secondary antibody (ProteinTech) at RT for 3 h and washed three times with TBST. The blots were developed by electrochemiluminescence (Advansta, San Jose, CA, USA) and imaged with the ChemiDoc MP imaging system (Bio-Rad, Hercules, CA, USA).

### 2.8. Luciferase Activity Assay

Wild-type (WT), mutant (MUT) lnc021545 3′-untranslated region (UTR), and EREG 3′-UTR were amplified and cloned into the luciferase reporter vector pmirGLO (Promega, Madison, WI, USA) to create pmirGLO lnc021545, pmirGLO EREG WT, and MUT plasmids. Then, 1 × 10^5^ MCF-7 cells were co-transfected with 5 ng each of the corresponding plasmids and 20 pmol each of miR-330-3p mimics. A dual-luciferase reporter assay system (Promega) was used in this assay. Each well of luminometer plates was loaded with lysates and the firefly luciferase activity was detected through an EnSpire multifunctional microplate reader (PerkinElmer, Waltham, MA, USA). Each well of the luminometer plates was again loaded with 100 µL Stop&Glo reagent, and renilla luciferase activity was detected. The results were represented as normalized firefly luciferase activity/renilla luciferase activity values.

### 2.9. Quantitative Reverse Transcription PCR (qRT-PCR) Assay

Total RNA was obtained by Trizol^TM^ reagent (Invitrogen). Then, 1 µg of total RNA was reversely transcribed into high-quality cDNA with PrimeScript^TM^ 1st cDNA Synthesis (Takara, Kyoto, Japan). qRT-PCR was performed using the FastStart Universal SYBR Green Master (Roche, Basel, Switzerland) on a StepOne^TM^ Real-Time PCR System (ABI, Los Angeles, CA, USA). The relative expression levels of EREG and lnc021545 were quantified using β-actin as the internal reference, while the relative expression levels of miR-330-3p were analyzed against U6 reference. The relative expression levels of RNA were calculated using the 2^−ΔΔCT^ method. The primer sequences are listed in Table S2.

### 2.10. Immunohistochemistry (IHC) Assay

The 2.5 μm paraffin slices were dewaxed by xylene, rehydrated with gradient ethanol, covered with 3% H_2_O_2_ for 10 min, washed with PBS three times, and incubated with goat anti-epiregulin polyclonal antibody (Invitrogen), rabbit anti estrogen receptor (ER) polyclonal antibody (Invitrogen), rabbit anti progesterone receptor (PR) monoclonal antibody (Invitrogen), or rabbit anti Ki-67 monoclonal antibody (Invitrogen) overnight at 4 °C. The slices were then nurtured with a biotin–streptavidin horseradish peroxidase detection system (ZSGB-BIO, Beijing, China) for 2 h and stained by DAB at RT. The slices were counterstained with hematoxylin, dehydrated with gradient ethanol, clarified with xylene, scanned with an Aperio GT450 (Leica, Wetzlar, Germany) at 200× magnification, and evaluated with an Aperio ImageScope (version 12.4.3.5008). The protein expression in the selected region was reflected by H-Score [20].

### 2.11. Fluorescence In Situ Hybridization (FISH)

The 3 μm paraffin slices were heated for 30 min at 56 °C. The slices were deparaffinized for 10 min with xylene and tapped off xylene for 5 s in 100% ethanol, repeated twice. The slides were placed into gradient ethanol and deionized water, then into the boiled pre-treatment solution for 15 min. The slices were washed with 2 × SSC solution, incubated with proteinase K solution for 15 min, washed with 2 × SSC solution for 1 min, and dehydrated with each gradient ethanol for 3 min at RT. The specimen area was dropped to a 10 μL probe mix. The coverslip was sealed with rubber cement, and the slices were hybridized at 47 °C overnight. The slices were cleaned with 2 × SSC solution and dehydrated in each gradient ethanol for 3 min. Following air-drying, the slides were counterstained with 10 μL DAPI counterstain. After 15 min, the slides were scanned with fluorescence microscope (Olympus) at 100× magnification.

### 2.12. Amplification-Refractory Mutation System PCR (ARMS-PCR) Assay

A standard pathology methodology was used to select the tumor tissue with more than 30% tumor cells for DNA extraction. Fifty BC patients’ DNA extractions were carried out from FFPE tumor tissue according to the instructions of the DNA extraction kit (AmoyDx, Xiamen, China). The qualities of DNA extractions were assessed by NanoDrop (Thermo Fisher, Waltham, MA, USA). Then, 5 ng sample DNA was added into each PCR reaction mix tube of PIK3CA mutations (AmoyDx). The PIK3CA mutations were detected on a 7500 Real-Time PCR System (ABI). The PIK3CA mutations were analyzed using the “Results Interpretation” analysis module.

### 2.13. Statistical Analysis

SPSS 22.0 (IBM, Armonk, NY, USA) and GraphPad Prism 7 (GraphPad, La Jolla, CA, USA) were used for data statistical analyses. The differences and statistical significance between the two groups of data were assessed using Student’s *t*-test. The relation between any two molecules in BC tissues was assessed using the Pearson correlation coefficient. *p* value < 0.05 was regarded as significant.

## 3. Results

### 3.1. Evaluation of the Effect of miR-330-3p and EREG in BC

**Figure 1:**
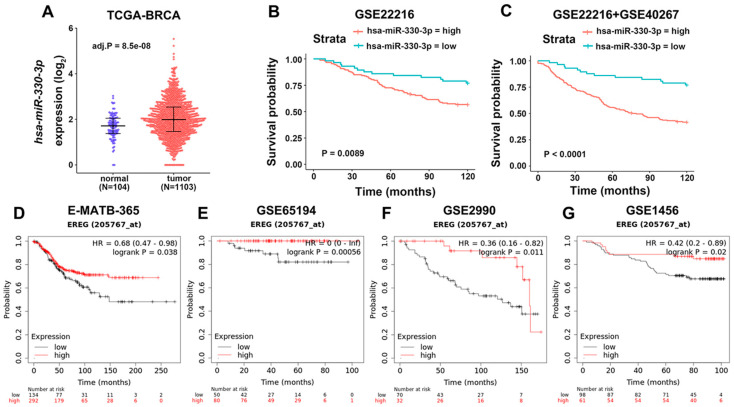
Evaluation of the roles of miR-330-3p and EREG in BC. (**A**) The expression level of miR-330-3p in BC specimens from the TCGA-BRCA dataset. (**B**,**C**) The expression level of miR-330-3p was associated with a poor clinical outcome from the GSE22216 and GSE40267 datasets. (**D**–**G**) EREG expression was negatively correlated with the clinical outcome of BC patients through analyzing the E-MATB-365, GSE65197, GSE2990, and GSE1456 datasets with the KMplot web tool.

To evaluate the role of miR-330-3p in BC, we performed an analysis of differential expression levels based on the TCGA-BRCA dataset. It showed that the expression of miR-330-3p was up-regulated in BC tissues compared to the normal controls (Figure 1A, *p* = 8.5 × 10^−8^). We further analyzed the association of miR-330-3p expression with the invasive disease-free survival (iDFS) of BC patients based on the GSE22216 dataset. Patients with low miR-330-3p expression showed better survival probability than patients with high miR-330-3p expression (Figure 1B, *p* = 0.0089). In addition, we pooled the BC samples from GSE22216 and GSE40267 into one metadata cohort including 265 patients who had undergone 10-year follow-up. The batch effect was removed using R package “sva”. We also stratified the iDFS by differential expression of miR-330-3p, resulting in a more significant survival analysis (Figure 1C, *p* < 0.0001). The data imply that the expression level of miR-330-3p might be a poor prognostic marker in BC.

To explore the effect of EREG in BC, we performed survival analysis using KMplot. We found that patients with high expression of EREG showed higher overall survival (OS) rates than patients with low expression of EREG from analyzing the E-MATB-365 (Figure 1D, *p* = 0.038), GSE65194 (Figure 1E, *p* = 0.00056), GSE2990 (Figure 1F, *p* = 0.011), and GSE1456 (Figure 1G, *p* = 0.02) datasets. The results suggest that EREG expression might be a positive prognostic marker in BC. The above results demonstrate miR-330-3p as a tumor promoter in BC and EREG as a tumor suppressor in BC. 

### 3.2. miR-330-3p Affects the Motility, Migration, and Invasion of BC Cells

**Figure 2:**
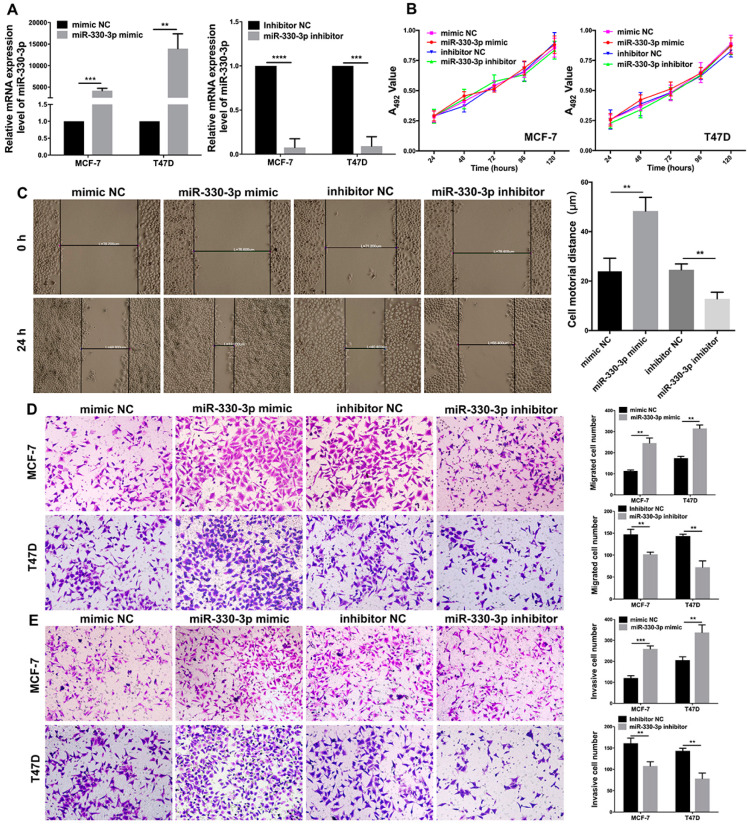
The influences of miR-330-3p on the proliferation, motility, migration, and invasion of BC cells. (**A**) In MCF-7 and T47D, the over-expression and knockdown of miR-330-3p were determined via qRT-PCR. (**B**) MTT assay was used to analyze the proliferations of MCF-7 and T47D cells. (**C**) The cell motilities of MCF-7 cells transfected with miR-330-3p mimic or inhibitor were measured via wound healing assay. The transwell assays were performed for cells’ (**D**) migrations and (**E**) invasions. (** *p* < 0.01, *** *p* < 0.001, **** *p* < 0.0001, *n* = 3).

MCF-7 and T47D cells were transiently transfected with miR-330-3p mimic, miR-330-3p inhibitor, or corresponding NC. The results of qRT-PCR showed that miR-330-3p expression was increased 4190-fold (*p* = 0.0002) in MCF-7 cells and 13,966-fold (*p* = 0.0043) in T47D cells in contrast with their corresponding NC group cells and was decreased by 92.2% (*p* < 0.0001) in MCF-7 cells and 90.7% (*p* = 0.0001) in T47D cells (Figure 2A).

MTT assay was used to assess the role of miR-330-3p on the proliferation in MCF-7 and T47D cells. Compared to their corresponding NC group cells, both the overexpression and downregulation of miR-330-3p had no effect on the proliferation of MCF-7 and T47D cells (*p* > 0.05, Figure 2B). This also suggests that the influence of miR-330-3p on the malignant behavior of BC without affecting its proliferation.

A wound healing assay was used to measure the effect of miR-330-3p on the motility of BC cells (Figure 2C). The scratch closure distance of MCF-7-miR-330-3p-mimic cells was measured as 48.3 ± 5.5 μm, which was 50.1% (*p* = 0.0052) more than that of MCF-7-mimic-NC cells. The scratch closure distance of MCF-7-miR-330-3p-inhibitor cells was 12.8 ± 2.7 μm, which was 48.0% (*p* = 0.0047) less than that of MCF-7-inhibitor-NC cells. Our data reveal that miR-330-3p overexpression promotes the motility of MCF-7 cells, while miR-330-3p downregulation had a converse effect. The results imply that miR-330-3p might positively affect the motility of BC cells.

The influences of miR-330-3p on the migrative and invasive abilities of BC cells were addressed using transwell assays. MiR-330-3p overexpression improved the migration and invasion of MCF-7 and T47D cells (Figure 2D,E). The numbers of migrated MCF-7-miR-330-3p-mimic (245.7 ± 23.8) and T47D-miR-330-3p-mimic (315.0 ± 16.7) cells were increased by 53.9% (*p* = 0.0083) and 44.8% (*p* = 0.0086) compared to those of the MCF-7-mimic-NC (113.2 ± 4.4) and T47D-mimic-NC cells (173.9 ± 8.7), respectively. The numbers of invaded MCF-7-miR-330-3p-mimic (259.3 ± 14.5) and T47D-miR-330-3p-mimic (338.0 ± 35.8) cells were increased by 53.6% (*p* = 0.0002) and 39.1% (*p* = 0.0091) compared to those of the MCF-7-mimic-NC (120.3 ± 11.5) and T47D-mimic-NC cells (206.0 ± 16.0), respectively. Consistently, miR-330-3p downregulation inhibited the migration and invasion of MCF-7 and T47D cells (Figure 2D,E). The numbers of migrated MCF-7-miR-330-3p-inhibitor (101.7 ± 5.0) and T47D-miR-330-3p-inhibitor (72.3 ± 14.4) cells were reduced by 32.4% (*p* = 0.0069) and 49.6% (*p* = 0.0071) compared to the MCF-7-inhibitor-NC (147.5 ± 11.5) and T47D-inhibitor-NC cells (143.4 ± 4.5), respectively. The numbers of invaded MCF-7-miR-330-3p-inhibitor (107.7 ± 10.0) and T47D-miR-330-3p-inhibitor (78.5 ± 12.9) cells were reduced by 33.1% (*p* = 0.01) and 45.3% (*p* = 0.0038) compared to MCF-7-inhibitor-NC (161.0 ± 12.1) and T47D-inhibitor-NC cells (143.5 ± 5.9), respectively. Our data reveal that miR-330-3p is a promoter of the migration and invasion of BC cells.

### 3.3. Lnc021545 Knockdown Promotes the Motility, Migration, and Invasion of BC Cells

**Figure 3:**
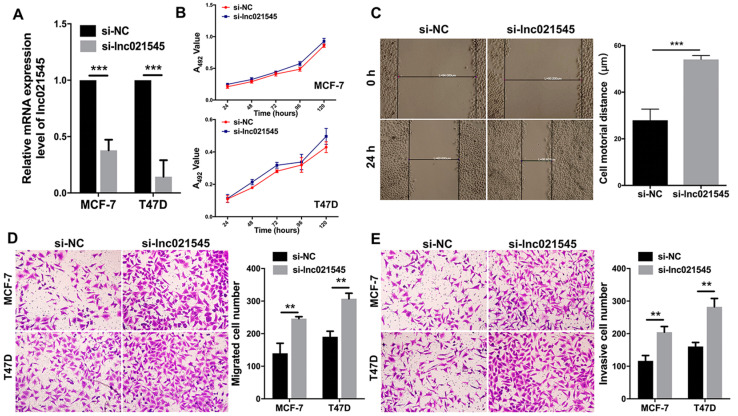
The influences of lnc021545 downregulations on the proliferation, motility, migration, and invasion of BC cells. (**A**) The downregulations of lnc021545 in MCF-7 and T47D cells were determined via qRT-PCR. (**B**) MTT assay was performed to measure the effect of lnc021545 knockdown on the proliferations of MCF-7 and T47D cells. (**C**) The effect of lnc021545 knockdown on the cell motility of MCF-7 cells was measured via wound healing assay. The transwell assays were performed for the cell (**D**) migration and (**E**) invasion. (** *p* < 0.01, *** *p* < 0.001, *n* = 3).

In MCF-7 and T47D cells, the lnc021545 expressions were downregulated by si-lnc021545 transient transfection. Compared with MCF-7-si-NC and T47D-si-NC cells, lnc021545 expression was reduced by 62.3% (*p* = 0.0003) and 85.7% (*p* = 0.0005) in MCF-7-si-lnc021545 and T47D-si-lnc021545 cells (Figure 3A), respectively.

MTT assay was measured the effect of lnc021545 on the proliferation of BC cells. Compared to the si-NC cells, the knockdown of lnc021545 showed no influence on the proliferation abilities of MCF-7 and T47D cells at 24 h, 48 h, 72 h, 96 h, and 120 h (*p* > 0.05, Figure 3B). This indicates that lnc021545 affects BC progression without affecting its proliferative capacities.

Comparative cell motility was demonstrated from cells’ movement via wound healing assay based on scratch closure distance. As shown in Figure 3C, a relatively higher scratch closure ratio was observed for si-lnc021545-transfected MCF-7 cells 24 h after wound scratches. The scratch closure distance of MCF-7-si-lnc021545 cells was measured as 54.0 ± 1.7 μm, which was 48.4% (*p* = 0.0009) more than that of MCF-7-si-NC cells. This indicates that lnc021545 downregulation might increase the motility of MCF-7 cells.

Boyden transwell assays were executed to investigate the effects of lnc021545 downregulation on the migration and invasion of MCF-7 and T47D cells. Lnc021545 knockdown increased their migration and invasion capacities (Figure 3D,E). The numbers of migrated MCF-7-si-lnc021545 (246.1 ± 3.5) and T47D-si-lnc021545 (307.3 ± 9.7) cells were increased by 43.3% (*p* = 0.0042) and 38.0% (*p* = 0.001) compared to their corresponding MCF-7-si-NC (139.4 ± 17.8) and T47D-si-NC cells (190.7 ± 9.7), respectively. The numbers of invaded MCF-7-si-lnc021545 (204.8 ± 9.8) and T47D-si-lnc021545 (282.3 ± 14.8) cells were increased by 43.1% (*p* = 0.003) and 43.1% (*p* = 0.0018) comparted to their corresponding MCF-7-si-NC (116.4 ± 16.6) and T47D-si-NC cells (160.7 ± 12.3), respectively. Clearly, lnc021545 significantly affected the migrative and invasive abilities of MCF-7 and T47D cells. It suggests that the downregulation of lnc021545 promotes the migration and invasion of BC cells in BC progression.

### 3.4. EREG Influences the Motility, Migration, and Invasion of BC Cells

**Figure 4:**
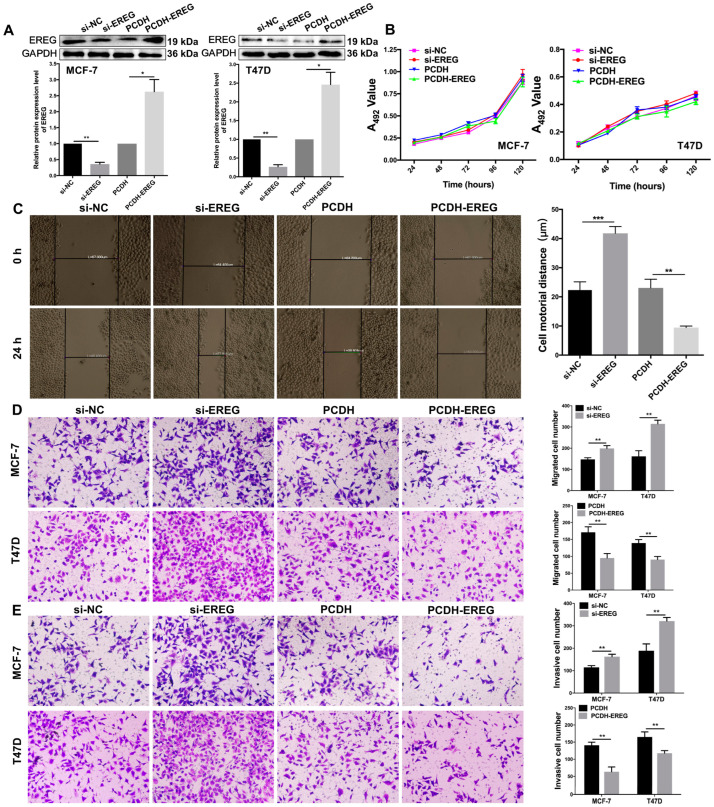
The influences of EREG on the proliferation, motility, migration, and invasion of BC cells. (**A**) The downregulation and overexpression of EREG in MCF-7 and T47D cells via WB assay. (**B**) MTT assays were performed for cell proliferations of MCF-7 and T47D cells. (**C**) The cell motility of MCF-7 cells was measured via wound healing assay. The transwell assays were performed for cells’ (**D**) migration and (**E**) invasion. (* *p* < 0.05, ** *p* < 0.01, *** *p* < 0.001, *n* = 3).

To explore the role of EREG in MCF-7 and T47D cells, si-NC, si-EREG, PCDH, or PCDH-EREG were transfected into them to downregulate and overexpress EREG. The WB assays revealed that the expression of EREG was reduced by 63.9% (*p* = 0.0023) in MCF-7 cells and 73.3% (*p* = 0.0019) in T47D cells and increased by 162.7% (*p* = 0.0177) in MCF-7 cells and 146.0% (*p* = 0.0166) in T47D cells compared to their respective NC cells (Figure 4A).

The effect of EREG on the proliferations of MCF-7 and T47D cells were assessed via MTT assay. The expression changes of EREG expression had no effect on the proliferations of either cell at 24 h, 48 h, 72 h, 96 h, and 120 h (*p* > 0.05, Figure 4B). These data suggest that EREG involves in BC malignancy without affecting BC cells’ proliferation.

As shown in Figure 4C, the scratch closure distance of MCF-7-si-EREG cells was 41.8 ± 2.3 μm, which was 46.7% (*p* = 0.0008) more than that of MCF-7-si-NC cells. Compared to the PCDH group, the scratch closure distance of MCF-7-PCDH-EREG cells was 9.4 ± 0.6 μm with a reduction of 59.3% (*p* = 0.0015). Therefore, the downregulation of EREG promotes the motility of MCF-7 cells, while EREG overexpression showed the opposite effect. Hence, EREG negatively affects the motility of BC cells.

The influences of EREG expression on the migration and invasion in MCF-7 and T47D cells were studied via transwell assays. EREG downregulation increased the migration and invasion of both cells (Figure 4D,E). The numbers of migrated MCF-7-si-EREG (199.0 ± 13.2) and T47D-si-EREG (314.7 ± 17.6) cells increased by 26.1% (*p* = 0.0042) and 48.7% (*p* = 0.0012) compared to MCF-7-si-NC (147.0 ± 7.8) and T47D-si-NC cells (161.3 ± 26.8), respectively. The numbers of invaded MCF-7-si-EREG (162.3 ± 10.7) and T47D-si-EREG (321.0 ± 16.1) cells were increased by 29.3% (*p* = 0.0032) and 41.2% (*p* = 0.0027) compared to MCF-7-si-NC (114.7 ± 7.5) and T47D-si-NC cells (188.7 ± 30.7), respectively. Oppositely, EREG overexpression reduced the migration and invasion of MCF-7 and T47D cells (Figure 4D,E). The numbers of migrated MCF-7-PCDH-EREG (95.3 ± 13.3) and T47D-PCDH-EREG (90.7 ± 9.1) cells were decreased by 44.3% (*p* = 0.0034) and 34.7% (*p* = 0.0038) compared to MCF-7-PCDH-NC (171.0 ± 16.4) and T47D-PCDH-NC cells (139.0 ± 10.5), respectively. The numbers of invaded MCF-7-PCDH-EREG (63.3 ± 14.0) and T47D-PCDH-EREG (117.7 ± 7.5) cells were decreased by 55.0% (*p* = 0.0013) and 28.7% (*p* = 0.008) compared to MCF-7-PCDH-NC (140.7 ± 9.1) and T47D-PCDH-NC cells (165.0 ± 14.9). These results show that EREG acts as a BC suppressor by inhibiting the migration and invasion of BC cells.

### 3.5. Lnc021545 Acts as a Sponge for miR-330-3p in BC Cells

**Figure 5:**
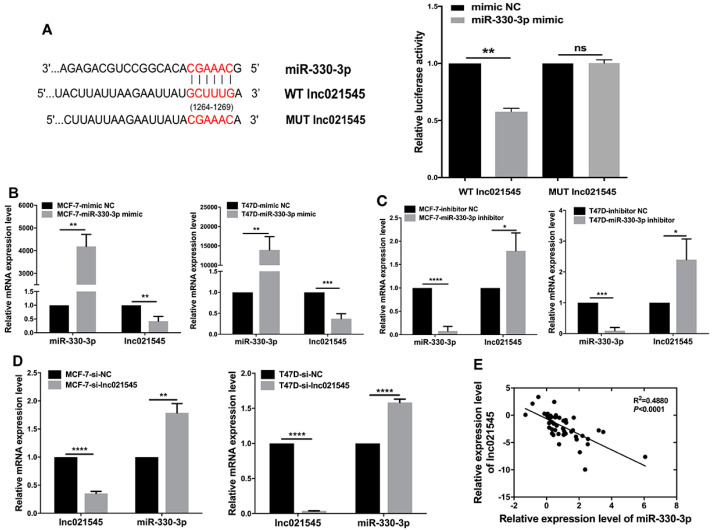
Lnc021545 acts as a sponge for miR-330-3p in BC cell. (**A**) The predicted and lucuferase activity assay proved binding sequences between miR-330-3p and lnc021545. Influences of miR-330-3p overexpression (**B**) and downregulation (**C**) on lnc021545 expression in BC cells. (**D**) Influence of lnc021545 downregulation on miR-330-3p expression in BC cells. (**E**) The interrelationship of miR-330-3p and lnc021545 expression changes in 50 BC patients’ specimens (ns: no significant different, * *p* < 0.05, ** *p* < 0.01, *** *p* < 0.001, **** *p* < 0.0001, *n* = 3).

To confirm the binding between miR-330-3p and lnc021545 in BC, we first used bioinformatic analysis to predict the potential binding of the 1264–1269 GCUUUG sequence at site of lnc021545 with the CGAAAC sequence of miR-330-3p (Figure 5A). To prove this point, we subcloned wild-type (WT lnc021545) and mutant-type (MUT lnc021545) miR-330-3p binding sites into the dual-luciferase reporters. As expected, co-transfection of miR-330-3p mimics substantially decreased the relative luciferase activity of the WT lnc021545 in MCF-7 cells (42.3%, *p* = 0.0017) but showed no effect on that of the MUT lnc021545 (Figure 5A), which suggests the direct binding of miR-330-3p with lnc021545.

To better understand the correlation between miR-330-3p and lnc021545, we transfected MCF-7 and T47D cells respectively with miR-330-3p inhibitor, miR-330-3p mimics, or si-lnc021545, and then observed the changes in lnc021545 expression or miR-330-3p expression. Accordingly, overexpressing miR-330-3p significantly decreased lnc021545 expression in MCF-7 (58.1%, *p* = 0.0043) and T47D cells (62.9%, *p* = 0.0008), whereas its knockdown increased lnc021545 expressions in MCF-7 (79.5%, *p* = 0.023) and T47D cells (140.0%, *p* = 0.0223) (Figure 5B,C). Similarly, lnc021545’s knockdown increased miR-330-3p expressions in MCF-7 (78.7%, *p* = 0.0012) and T47D cells (58.5%, *p* < 0.0001) (Figure 5D). The expression level changes of miR-330-3p and lnc021545 expression show a negative correlation in BC cells.

To further verify the negative correlation between miR-330-3p and lnc021545, we analyzed the relationship between miR-330-3p and lnc021545 in BC patients’ tissues. Our previous results revealed that in 50 paired BC patients’ samples, miR-330-3p expression was notably increased and lnc021545 expression was the opposite, and we observed a significantly negative association between miR-330-3p expression and lnc021545 expression (R2 = 0.4880, *p* < 0.0001, Figure 5E). Lnc021545 deregulation is involved in BC carcinogenesis by reversely mediating the expression of miR-330-3p.

### 3.6. miR-330-3p Expression Is Negatively Correlated with EREG Expression in BC

**Figure 6:**
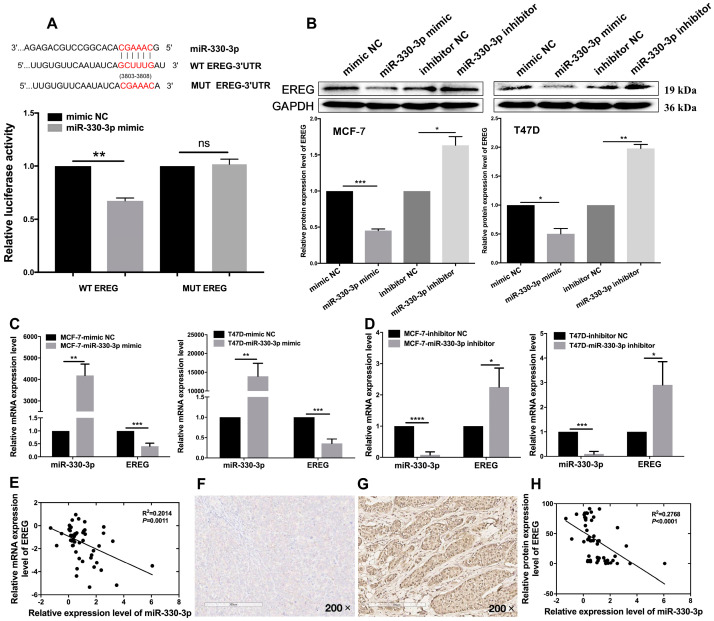
EREG directly interacts with miR-330-3p in BC. (**A**) Dual-luciferase reporter assay validates the predictive binding sites between miR-330-3p and EREG. (**B**) In MCF-7 and T47D cells, the effects of miR-330-3p overexpression and downregulation on EREG protein expression. In MCF-7 and T47D cell lines, effects of miR-330-3p overexpression (**C**) and miR-330-3p downregulation (**D**) on EREG mRNA expression. (**E**) The correlation analysis of changes of miR-330-3p and EREG in 50 BC patients via qRT-PCR assay. The representative IHC images of patients’ specimens with low (**F**) and high (**G**) EREG abundance based on the median of H-score of EREG protein expression. (**H**) The correlation analysis of miR-330-3p and EREG protein expressions level changes in 50 BC patients. (ns: no significant difference, * *p* < 0.05, ** *p* < 0.01, *** *p* < 0.001, **** *p* < 0.0001, *n* = 3).

To demonstrate that miR-330-3p binds to EREG in BC, we first predicted that the GCUUUG at 3,803–3,808 site of EREG-3′-UTR might complementarily bind to the CGAAAC sequence of miR-330-3p (Figure 6A). To verify whether EREG was a target gene of miR-330-3p in BC, through the dual-luciferase reporter assay, we found that miR-330-3p mimic remarkably decreased the relative luciferase activity of the WT EREG (32.7%, *p* = 0.002) in MCF-7 cells but did not affect that of the MUT EREG (Figure 6A). These data provide evidence that miR-330-3p can directly bind to EREG-3′-UTR.

The changes in EREG expression following transfection with miR-330-3p mimic or inhibitor in MCF-7 and T47D cells were checked using qRT-PCR and WB assays. The results as shown in Figure 6B–D demonstrated that overexpression of miR-330-3p downregulated the mRNA and protein levels of EREG in MCF-7 (59.1%, *p* = 0.0009, 54.7%, *p* = 0.0005) and T47D (65.5%, *p* = 0.0005, 49.8%, *p* = 0.0110) cells. Accordingly, miR-330-3p knockdowns increased endogenous EREG expression. The EREG mRNA and protein levels were increased in MCF-7 (124.8%, *p* = 0.0242, 63.3%, *p* = 0.0114) and T47D (190.7%, *p* = 0.0249, 98.8%, *p* = 0.0016) cells (Figure 6B–D). The data indicated that miR-330-3p reversely mediates EREG expression by targeting the latter’s 3-UTR in BC cells.

The negative correlation of EREG expression alteration with miR-330-3p differential expression was greater in 50 paired BC patients’ tissues (R^2^ = 0.2014, *p* = 0.0011, Figure 6E). Furthermore, we investigated the EREG expression profiles in the above-mentioned tissues by IHC. In the previous experiments, these patients have already been detected for lnc021545 expression and miR-330-3p expression. MiR-330-3p and EREG differential expression changes were allocated to low and high expression groups based on the median H-score of EREG protein expression (Figure 6F,G). The expression alteration of miR-330-3p showed an apparent negative correlation with EREG protein expression alteration (R^2^ = 0.2768, *p* < 0.0001, Figure 6H). Altogether, our data declare that EREG is a directly downstream goal of miR-330-3p via binding to its 3′-UTR to negatively mediate EREG expression in BC.

### 3.7. Lnc021545/miR-330-3p Axis Affects BC Metastasis by Regulating EREG Expression

**Figure 7:**
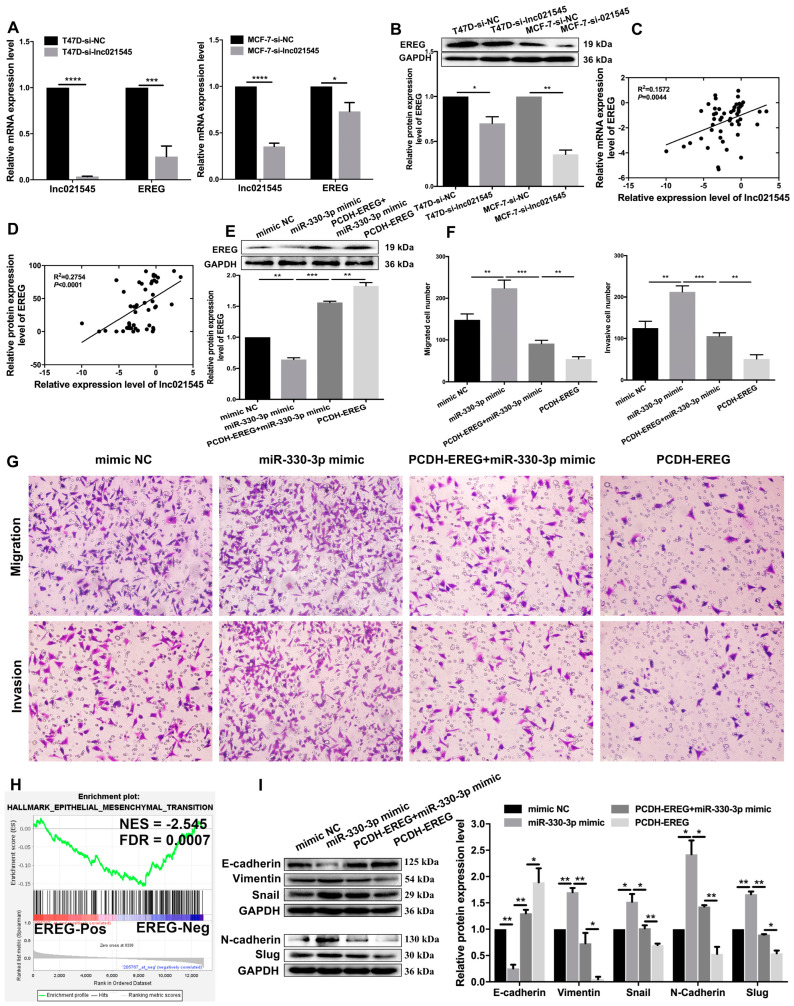
Lnc021545/miR-330-3p regulates BC metastasis by affecting the expression of EREG. Effects of knockdown lnc021545 on EREG mRNA (**A**) and EREG protein (**B**) in T47D and MCF-7 cells. Correlation between lnc021545 and EREG mRNA (**C**) and EREG protein (**D**) in 50 BC tissues. In the rescue experiment, EREG protein expression level was detected using a WB assay (**E**); migration and invasion of MCF-7 cell line were measured using a transwell assay (**F**,**G**). Single-gene GSEA showing the association between EREG and EMT based on GSE3494 dataset (**H**). The expression levels of EMT markers were measured using WB assays in the rescue experiment (**I**) (* *p* < 0.05, ** *p* < 0.01, *** *p* < 0.001, **** *p* < 0.0001, *n* = 3).

To verify that lnc021545 can affect free miR-330-3p and then regulate EREG expression, we explored the effect between downexpression of lnc021545 and EREG. Lnc021545 downregulation decreased endogenous EREG expressions. EREG mRNA and protein expression were reduced by 74.8% (*p* = 0.0003) and 29.9% (*p* = 0.0202), respectively, in T47D cells and by 26.9% (*p* = 0.0127) and 64.3% (*p* = 0.0018), respectively, in MCF-7 cells following lnc021545 knockdown in them (Figure 7A,B).

To further confirm the correlations between lnc021545 and EREG, we analyzed the relationship between lnc021545 and EREG expression level changes in BC tissues. We observed that lnc021545 had a positive relationship with EREG mRNA expression (R^2^ = 0.1572, *p* = 0.0044, Figure 7C) and the protein expression level of EREG (R^2^ = 0.2754, *p* < 0.0001, Figure 7D).

To establish the effects of the lnc021545-miR-330-3p-EREG axis in BC cells, we co-transfected miR-330-3p mimic and PCDH-EREG plasmid in MCF-7 cells. Compared with the MCF-7 cells transfected with mimic NC, EREG expression was decreased by 35.5% (*p* = 0.002) compared to that of the cells transfected with miR-330-3p mimic. In the co-transfected PCDH-EREG and miR-330-3p mimic cells, EREG expression was 91.7% (*p* = 0.0006) more than that of the cells transfected with miR-330-3p mimic and 26.8% (*p* = 0.0051) less than that of the cells transfected with PCDH-EREG. The results indicate that MCF-7 cells co-transfected with miR-330-3p mimic and PCDH-EREG partially restored inhibition of EREG expression in MCF-7 cells transfected with miR-330-3p mimic (Figure 7E).

A similar phenomenon was perceived in transwell assay experiments (Figure 7F,G). The number of migrated MCF-7 cells co-transfected with PCDH-EREG and miR-330-3p mimic (91.2 ± 8.0) was 59.3% (*p* = 0.0004) less than that of MCF-7 cells transfected with miR-330-3p mimic (224.1 ± 19.4) and 39.8% (*p* = 0.0029) more than that of MCF-7 cells transfected with PCDH-EREG (54.8 ± 5.5). The number of invaded MCF-7 cells co-transfected with miR-330-3p mimic and PCDH-EREG (105.5 ± 8.0) were 50.3% (*p* = 0.0004) less than that of MCF-7 cells transfected with miR-330-3p mimic (212.4 ± 14.5) and 51.7% (*p* = 0.0019) more than that of MCF-7 cells transfected with PCDH-EREG (50.9 ± 10.2). The rescue experiment evidence indicates lnc021545-miR-330-3p axis functionalizing in BC carcinogenesis through regulating the expression of endogenous EREG in BC cells.

To clarify the mechanism of lnc021545-miR-330-3p-EREG axis plays in BC metastasis, we recognized the extraordinarily regulated functional gene sets via single-gene GSEA. It showed a significantly negative correlation between EREG (NES = −2.455, FDR = 0.0007) and EMT (Figure 7H) process. Further analysis of the rescue experiment showed that compared to MCF-7 cells co-transfected with PCDH-EREG and miR-330-3p mimic, MCF-7 cells transfected with miR-330-3p mimic significantly reduced E-cadherin protein expression (104.8%, *p* = 0.0057) and significantly promoted vimentin (97.0%, *p* = 0.0048), Snail (50.3%, *p* = 0.018), N-cadherin (99.3%, *p* = 0.0266), and Slug (76.8%, *p* = 0.001) protein expressions. Meanwhile, MCF-7 cells treated with PCDH-EREG significantly increased the expression of E-cadherin (58.9%, *p* = 0.038) and significantly decreased the expressions of vimentin (68.3%, *p* = 0.017), Snail (32.0%, *p* = 0.0056), N-cadherin (89.9%, *p* = 0.0047), and Slug (35.6%, *p* = 0.0103) (Figure 7I). These data indicate that the lnc021545-miR-330-3p axis mediated EMT process to inhibit the metastasis of BC cells via specifically suppressing EREG expression, and it verified that the effect of the lnc021545-miR-330-3p axis in repressing the metastasis of BC primarily depends on the EREG to influence the changes of EMT markers.

### 3.8. The Clinical Feature and Cooperative Effects of lnc021545, miR-330-3p, and EREG in BC Patients

**Figure 8:**
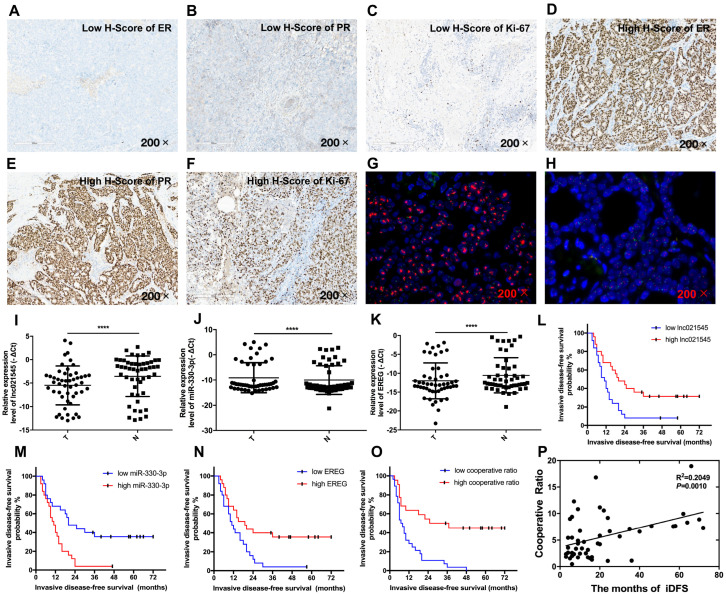
The clinical feature and cooperative effects of lnc021545, miR-330-3p, and EREG in BC patients. The images of BC patients’ specimens with low H-scores of ER (**A**), PR (**B**), and Ki-67 (**C**). The images of BC patients’ specimens with high H-scores of ER (**D**), PR (**E**), and Ki-67 (**F**). The images of BC patients’ specimens with HER-2 amplification (**G**); the images of BC patients’ specimens with HER-2 un-amplification (**H**). The expression level analyses of lnc021545 (**I**), miR-330-3p (**J**), and EREG (**K**) in adjacent non-tumor tissues and BC tissues. Kaplan–Meier curve of iDFS in the expression of lnc021545 (**L**), miR-330-3p (**M**), and EREG (**N**). Kaplan–Meier curve of iDFS in the cooperative ratio (**O**). Correlation between cooperative ratio and months of iDFS in 50 BC tissues (**P**). (**** *p* < 0.0001, *n* = 50).

The relative expressions of miR-330-3p, lnc021545, and EREG were already measured in 50 BC patients’ tissues using the qRT-PCR assay mentioned above. Moreover, we underwent 7 years of follow-up for 50 patients and stratified clinical pathological features from them through expressions of miR-330-3p, lnc021545, and EREG. Fifty patients were allocated to different expression levels according to the median of the three molecules expressions in BC tissues to establish the connection of clinicopathologic features and prognosis of BC. Fifty patients were allocated to different expression levels according to the H-score medians of ER, PR, and Ki-67 protein expression by IHC (Figure 8A–F), and the three molecules are all expressed in the nucleus. The median of the H-scores of ER, PR, and Ki-67 respectively 137.1, 125.0, and 88.1, respectively. Fifty BC patients were allocated to the groups of HER-2 amplification and HER-2 un-amplification based on the results of FISH assay (Figure 8G,H). Accordingly, 15 out of 50 BC patients had HER-2 amplification, while the remaining 35 patients had HER-2 un-amplification.

Lnc021545 was significantly downregulated in 50 BC patients’ cancerous tissues (*p* < 0.0001, Figure 8I). We found that low expression of lnc021545 was closely associated with some clinical features (Table 1)—for example, tumor size (*p* = 0.0025), advanced TNM stage (*p* = 0.003), lymph node metastasis (*p* = 0.01), and HER-2 amplification (*p* = 0.012). The results indicated that the reduced expression of lnc021545 is detected in BC and may be involved in the progression and trastuzumab therapy of BC. In these clinical data, we also found that lnc021545 was not related to age; position; vascular invasion; nerve invasion; the expression of ER, PR, or Ki-67; or the mutant of PIK3CA (*p* > 0.05). To investigate lnc021545’s prognostic value, we analyzed the effects of lnc021545 status on iDFS in 50 BC patients. The iDFS of cases with high lnc021545 expression were better than those of cases with low lnc021545 expression (*p* = 0.0085, Figure 8L). These results implicate that lnc021545 plays a suppressor role in BC.

MiR-330-3p expression was markedly increased in BC tumorous tissues (*p* < 0.0001, Figure 8J). MiR-330-3p was also closely associated with some clinical features (Table 1). High expression of miR-330-3p was related to the tumor size (*p* < 0.0001), advanced TNM stage (*p* < 0.0001), lymph node metastasis (*p* = 0.001), pathological stage (*p* = 0.012), and HER-2 amplification (*p* = 0.001). MiR-330-3p overexpression was positively associated with the progression and trastuzumab therapy of BC. Moreover, high miR-330-3p expression resulted in a poorer iDFS of BC patients (*p* = 0.0016, Figure 8M). The result suggests that miR-330-3p is a negative tumor marker for BC.

EREG expression was significantly reduced in 50 cancerous tissues (*p* < 0.0001, Figure 8K). As shown in Table 1, low EREG expression was associated with the tumor size (*p* < 0.0001), advanced TNM stage (*p* = 0.003), lymph node metastasis (*p* = 0.045), HER-2 amplification (*p* = 0.001), and ER expression (*p* = 0.023). Our data reveal that EREG normal expression may play a positive effect in inhibiting the progression and trastuzumab therapy in BC. Moreover, high EREG expression had a longer iDFS than a low EREG expression (*p* = 0.0099, Figure 8N) in BC patients and a better prognostic. The above results suggest that EREG might be a good prognostic factor of BC by inhibiting BC metastasis.

To explore whether lnc021545, miR-330-3p, and EREG work together to regulate the malignancy of BC, a cooperative ratio introduced by us as the sum of the relative expressions of lnc021545 and EREG divided by the relative expression of miR-330-3p was applied to address the cooperative prediction utilization of the three molecules with certain clinicopathologic features of BC. Interestingly, the introduction of this parameter led us to notice a low cooperative ratio was associated with tumor size (*p* = 0.05), advanced TNM stage (*p* = 0.008), lymph node metastasis (*p* = 0.035), and HER-2 amplification (*p* = 0.021), as shown in Table 1. The data declare that the cooperative ratio can be used to speculate on the progression and trastuzumab therapy of BC. A lower cooperative ratio resulted in a poorer iDFS (*p* < 0.0001, Figure 8O) and a shorter survival duration in month of iDFS (Figure 8P, R^2^ = 0.2049, *p* = 0.001) for BC patients. In our study, we observed that 10 deaths all occurred at a low cooperative ratio, a low expression of lnc021545, or a high expression of miR-330-3p, while only 6 deaths occurred at a low expression of EREG. More interestingly, recurrence occurred in all the patients cataloged with low cooperative ratios, while the patients without recurrence were either characterized with low lnc021545 expression, with high miR-330-3p expression, or with low EREG expression, which suggests that the integrated comparative utilization of low expressions of lnc021545 and EREG and high expression of miR-330-3p more accurately assess the prognosis of patients than any single factor.

## 4. Discussion

BC has become a significant health problem in modern society [1]. In the past few years, the metastasis and poor prognosis of BC have presented a great challenge despite the progression of chemotherapy, endocrine therapy, and HER-2-targeted therapy [21].

Lymphatic metastasis is the most important mode by which BC spreads, mainly including axillary lymphatic metastasis and parasternal lymphatic metastasis [5]. The management of lymphatic metastasis has become an important link in the treatment of BC. Therefore, the research on lymphatic metastasis of BC has become an important part of the diagnosis and treatment of BC. In previous research, the high-throughput genetic sequencing assays using two murine hepatocarcinoma cell lines with different lymphatic metastasis potentials from our group (unpublished) prompted us to believe that miR-330-3p, lnc021545, and EREG might also form a regulation mechanism in human cancers. Through analyzing the TCGA-BRCA dataset, we noticed that miR-330-3p was overexpressed in BC tumorous tissues. Patients with low expression of miR-330-3p showed longer iDFS probabilities than those with high expression of miR-330-3p. We used KMplot to analyze the association between EREG expression and BC patients’ OS. We found that patients with high EREG expression showed better OS probabilities than the patients with low EREG expression. As a novel lncRNA, profile of lnc021545 in BC is unavailable. Herein, its role in BC was not analyzed. From the analysis of the database, it can be seen that miR-330-3p might be a tumor promoter and that EREG might be a tumor suppressor in BC. We herein investigated their mutual relationship and their correlation together with their function mechanism and potential clinical practice in BC metastasis.

MiRNAs play important roles in the progressions and treatments of multiple tumors [22]. Earlier research has confirmed that miR-330-3p acted as a tumor promoter in multiple cancers. miR-330-3p acted as a tumor promoter in multiple cancers. It accelerated the migration and EMT of NSCLC cells via GGRIA3-TGFβ1 [23]. In hepatocellular carcinoma, miR-330-3p enhanced cancer cells’ viability, migration, invasion, and apoptosis resistance by binding to BTG1 [24]. MiR-330-3p targeted the CCBE1 and reduced its expression to promote the metastasis of BC [25]. MiRNAs have multiple target genes and affect the progression of diseases by regulating the interaction of multiple genes, even in the same disease. For example, miR-200 activates the EMT by targeting HIPK1 or HDAC2 to promote the metastasis of BC [26,27]. Our study aims to demonstrate not only the role of miR-330-3p in BC metastasis but also its correlations with lnc021545 and EREG together with their axial regulation mechanism in BC metastasis.

LncRNAs are a type of new-style biomarkers and potential therapeutic targets in mediating BC progression and metastasis through regulating gene expression [28,29]. Currently, the research on lnc021545 in disease is limited. We found that lnc021545 knockdown promoted the metastatic abilities of BC cells rather than altering their proliferations. As a member of the EGR family, EREG can bind to each of EGFR and ErbB-4 through ligand-induced heterodimerization, which affects the progression of diseases through activating signaling pathways downstream of ErbB1/ErbB [30]. EREG has largely been researched in the prognosis and drug resistance of BC [31], but little is known about its action mechanism in BC progression. In BC cells, EREG also reduced the tendency of metastasis but had no effect on the proliferation. Therefore, lnc021545 and EREG deficiency promotes the progression of BC by increasing the metastatic behaviors of BC cells.

We discovered that the changes in the three molecules’ expression levels consistently conferred greater metastasis of BC cells rather than altering proliferation. We also found this phenomenon in BC tumorous samples; there was no correlation between any of the three molecules and the expression of Ki-67, which is a marker of cell proliferation and reflects the degree of proliferation in BC. Consequently, these data suggest that the three molecules regulate BC patients’ outcomes and treatment through mediating the metastasis of BC cells.

In recent studies, the association between miRNAs and lncRNA has been the most popular as lncRNAs can bind to miRNAs to antagonize miRNA-induced activity [32]. We found that lnc021545 sponges miR-330-3p in BC cells. Bioinformatic analysis and dual-luciferase reporter assay demonstrated that lnc021545 targeted miR-330-3p to inhibit the latter’s expression. Our study also provided the relationship between lnc021545 and miR-330-3p in BC cells. Lnc021545 knockdown resulted in miR-330-3p upregulation, and the change in lnc021545 or miR-330-3p expression resulted in the corresponding change in miR-330-3p or lnc021545 expression in BC cells. MiR-330-3p and lnc021545 expression with a negative relationship was shown in the clinical data of 50 BC patients. By binding to each other to reversely regulate the expression of the other one, lnc021545 and miR-330-3p are functionalized in the metastasis of BC.

In order to prove that lnc021545, miR-330-3p, and EREG conform to this regulatory mode to modulate the metastasis of BC, we proved that lnc021545 could combine with miR-330-3p to affect BC’s metastasis in previous experiments, and we still need to prove that miR-330-3p and EREG could combine and interact with each other. Therefore, we proved that miR-330-3p could directly bound to 3′-UTR of EREG by using the online target gene prediction tool and the dual-luciferase reporters’ assay. Meanwhile, EREG expression levels was remarkably reversely regulated by the expression level change of miR-330-3p upregulated in BC cells. Further analysis confirmed that the mRNA and protein expression of EREG had a significant negative correlation with miR-330-3p in BC patients. We further showed that EREG mRNA and protein levels had an obviously positive association with lnc021545. Subsequently, EREG expression was remarkably suppressed in si-lnc021545 transfected cells. Our data demonstrate that lnc021545, miR-330-3p, and EREG are closely correlated in regulating the BC’s metastasis.

Cumulative evidence has pointed out that lncRNAs could modulate the biological activity of mRNA via hindering the miRNA from binding to its target mRNAs [33]. Although we failed to overexpress lnc021545 in BC cells, its knockdown in BC cells still clearly led us to detect the increase of miR-330-3p expression and the decrease of EREG expression. The overexpression or knockdown of miR-330-3p level led to reduced or increased expressions of lnc021545 and EREG in BC cells. miR-330-3p was consistently inversely related to lnc021545 and EREG in BC tissues. EREG was positively related to lnc021545 in BC tissues. Meanwhile, we also confirmed that miR-330-3p had direct bindings between lnc021545 and EREG. In summary, lnc021545, miR-330-3p, and EREG form a negative feedback regulation pathway in promoting BC. Nevertheless, the functions and biological mechanism of the lnc021545-miR-330-3p-EREG axis in the metastasis of BC are unclear.

**Figure 9:**
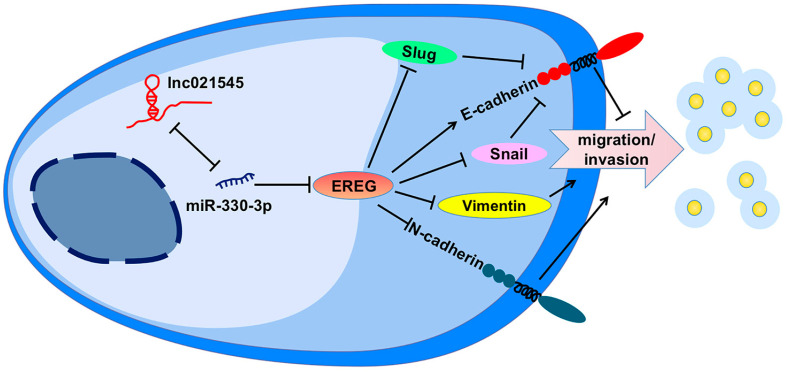
A schematic action mechanism of the lnc021545-miR-330-3p-EREG axis in the BC’ malignancy.

EMT has been demonstrated as an essential driver of carcinoma metastasis [34,35,36]. Growing evidence has proved that miRNAs regulated the EMT by interacting with certain targeted mRNAs to impact carcinoma metastasis. miR-508-3p hindered the EMT by targeting ZEB1 to inhibit cell invasion in triple-negative breast cancer [37]. Noyan et al. demonstrated that miR-770-5p targeted DNMT3A to suppress the EMT and the invasion of triple-negative breast cancer cells [38]. In earlier research, we confirmed EREG was one of the downstream target genes of miR-330-3p. Specifically, we confirmed that miR-330-3p could reduce endogenous EREG protein expression to promote and restore the metastasis of BC cells. Their mechanisms for the changes in metastasis of BC were elusive. Single-gene GSEA database analysis indicated EREG had a negative correlation with EMT. We consistently detected that miR-330-3p overexpression increased the expressions of mesenchymal molecules vimentin and N-cadherin, repressors of the epithelial molecules Snail and Slug, but decreased the expression of the epithelial molecule E-cadherin in MCF-7 cells via directly targeting EREG. These data confirm that lnc021545 affects the metastasis of BC by inhibiting miR-330-3p regulation on EREG expression through EMT process (Figure 9).

After we verified the mechanism by which the three molecules affect the metastasis of BC, we further observed the clinical effect of the three molecules on the therapeutic outcome and prognosis of BC patients. In the process of individualized treatment of BC, the expression of ER and PR, amplification of HER-2, and mutation of PIK3CA are some treatment-related indicators [39,40]. ER and PR expression levels correlate with endocrine therapy in BC and are the indexes used to develop therapeutic strategy and its efficacy [39]. The detection of HER-2 amplification is very crucial for the prognosis and treatment of BC; the amplification of the HER-2 gene suggests that BC patients have a high degree of malignancy and poor prognosis, which is suitable for targeted therapy [39]. Mutations of PIK3CA are one of the most common oncogenic mutations that occur in BC patients who may benefit from the treatments with PI3K inhibitors [39].

In 50 BC patients, we observed that miR-330-3p expression was elevated in cancerous tissues and related to tumor size, TNM stage, lymph node metastasis, pathological stage, and HER-2 amplification while lnc021545 and EREG were decreased in BC tissues and exerted the opposite effects of miR-330-3p in the BC patient cohorts. We also established that high expression of miR-330-3p had a shorter iDFS and high expression of lnc021545 and EREG were conducive to lengthening BC patients’ iDFS. All of these suggest that miR-330-3p overexpression or lnc021545 or EREG downregulation contribute to the progression of BC and a poor prognosis while raising the chances for BC patients to benefit from trastuzumab therapy.

In summary, we found that miR-330-3p, lnc021545, and EREG had links to the prognosis and treatment of BC, but there were some differences among the three molecules. The process of tumor requires the cooperation of multiple genes [16]. Therefore, we further analyzed the above results and tried to find an expressive mode to more accurately reflect the synergistic effect of the three molecules on the progression and treatment of BC. We established the expressive mode of the cooperative ratio according to the relationship and functions of the three molecules. Interestingly, we found that the patients with a low cooperative ratio had a more aggressive malignancy in BC patients, and the cooperative ratio was correlated with the size of the tumor, pathological TNM stage, lymphatic metastasis, pathological stage, and HER-2 amplification. It was concluded that the cooperative ratio had a good directivity for the malignant evaluation and clinical treatment of BC. More interestingly, the high cooperative ratio restrains the progression of BC and reduces the chance of trastuzumab therapy. Moreover, we also observed that a high cooperative ratio was conducive to lengthening BC patients’ iDFS, and the cooperative ratio was correlated with the months of iDFS. The results of these two parts showed that the cooperation ratio was closely tried with BC patients’ prognosis. Importantly, we found that the cooperative ratio of the three molecules was more comprehensive than each single molecule in the assessment of the patient’s malignant grade, recurrence, and death. Current work indicates that the roles and synergistic cooperation of lnc021545, miR-330-3p, and ERGE in regulating the malignant behaviors of BC cells as well as in evaluating the progression, therapeutic treatment, and prognosis of BC patients.

## 5. Conclusions

Lnc021545 inhibits miR-330-3p expression to modulate EREG expression, thereby affecting BC metastasis through mediating EMT. Our results highlight the coordinated functions of lnc021545, miR-330-3p, and EREG in BC progression, metastasis, and therapy. The lnc021545-miR-330-3p-EREG axis may act as a pivotal role in BC and even in other cancers. The combinational utilization of the three molecules may serve as a more accurate indicator for the carcinogenesis and a more precise target for the anti-metastasis therapeutic and prognosis of BC.

## Figures and Tables

**Table 1:** Correlations of miR-330-3p, lnc021545, and EREG and their cooperative ratio with the clinicopathological features of 50 BC patients.

Parameters	Group	lnc021545			miR-330-3p			EREG			Cooperative Ratio		
		Low	High	*p*	Low	High	*p*	Low	High	*p*	Low	High	*p*
**Age**	>60	11	12	1	10	13	0.571	12	11	1	14	9	1
	≤60	14	13		15	12		13	14		16	11	
**Position**	left	13	11	0.778	11	13	0.778	13	11	0.778	15	11	0.779
	right	12	14		14	12		12	14		15	9	
**Size**	<5 cm	14	22	0.025	25	10	0.000	12	24	0.000	19	18	0.05
	≥5 cm	11	3		1	14		13	1		11	2	
**TNM stage**	I–II	10	21	0.003	25	6	0.000	10	21	0.003	14	17	0.008
	III–IV	15	4		0	19		15	4		16	3	
**LN metastasis**	absence	6	16	0.01	17	5	0.001	7	15	0.045	9	13	0.021
	presence	19	9		8	20		18	10		21	7	
**Pathological stage**	II	16	19	0.538	22	13	0.012	17	18	1	19	16	0.345
	III	9	6		3	12		8	7		11	4	
**Vascular invasion**	absence	13	16	0.567	16	13	0.567	14	15	1	17	12	1
	presence	12	9		9	12		11	10		13	8	
**Nerve invasion**	absence	20	21	1	19	22	0.463	20	21	1	26	16	0.697
	presence	5	4		6	3		5	4		4	4	
**ER**	high	11	14	0.572	16	9	0.089	8	17	0.023	12	12	0.248
	low	14	11		9	16		17	8		18	8	
**PR**	high	10	16	0.156	16	10	0.156	14	12	0.778	15	10	1
	low	15	9		9	15		11	13		15	10	
**HER-2 amplification**	positive	12	3	0.012	2	13	0.001	13	2	0.001	14	1	0.002
	negative	13	22		23	12		12	23		16	19	
**Ki-67**	high	12	6	0.140	6	12	0.140	10	8	0.769	13	5	0.237
	low	13	19		19	13		15	17		17	15	
**PIK3CA**	mutant	7	10	0.551	10	7	0.551	8	9	1	11	6	0.763
	wild	18	15		15	18		17	16		19	14	

## Data Availability

The raw data supporting the conclusions of this article will be made available by the authors without undue reservation.

## Supplementary Materials

The following supporting information can be downloaded at: https://www.mdpi.com/article/10.3390/jcm12072478/s1, Table S1: Sequences of si-RNAs for EREG and lnc021545 knockdown; Table S2: Sequences of primers and oligonucleotides.

## References

[1] Sung H., Ferlay J., Siegel R.L., Laversanne M., Soerjomataram I., Jemal A., Bray F. (2021). Global cancer statistics 2020: GLOBOCAN estimates of incidence and mortality worldwide for 36 cancers in 185 countries, 2021. CA Cancer J. Clin..

[2] Gonzalez-Angulo A.M., Morales-Vasquez F., Hortobagyi G.N. (2007). Overview of resistance to systemic therapy in patients with breast cancer. Adv. Exp. Med. Biol..

[3] Park M., Kim D., Ko S., Kim A., Mo K., Yoon H. (2022). Breast cancer metastasis: Mechanisms and therapeutic implications. Int. J. Mol. Sci..

[4] Zhang Y., Weinberg R.A. (2018). Epithelial-to-mesenchymal transition in cancer: Complexity and opportunities. Front. Med..

[5] Li K., Li G.D., Sun L.Y., Li X.Q. (2018). PTEN and SHIP: Impact on lymphatic metastasis in breast cancer. J. Cancer Res. Ther..

[6] Liu B., Li J.Y., Cairns M.J. (2014). Identifying miRNAs, targets and functions. Brief Bioinform..

[7] Liu J., Liu L., Chao S., Liu Y., Liu X., Zheng J., Chen J., Gong W., Teng H., Li Z. (2017). The role of miR-330-3p/PKC-α signaling pathway in low-dose endothelial-monocyte activating polypeptide-II increasing the permeability of blood-tumor barrier. Front. Cell Neurosci..

[8] Jafarzadeh A., Paknahad M.H., Nemati M., Jafarzadeh S., Mahjoubin-Tehran M., Rajabi A., Shojaie L., Mirzaei H. (2022). Dysregulated expression and functions of microRNA-330 in cancers: A potential therapeutic target. Biomed. Pharmacother..

[9] Schmitz S.U., Grote P., Herrmann B.G. (2016). Mechanisms of long noncoding RNA function in development and disease. Cell. Mol. Life Sci..

[10] Smillie C.L., Sirey T., Ponting C.P. (2018). Complexities of post-transcriptional regulation and the modeling of ceRNA crosstalk. Crit Rev. Biochem. Mol. Biol..

[11] Cheng W., Feng P., Lee K., Chen K., Sun W., Van Hiep N., Luo C., Wu S. (2021). The role of EREG/EGFR pathway in tumor progression. Int. J. Mol. Sci..

[12] Roy S., Khanna S., Rink C., Biswas S., Sen C.K. (2008). Characterization of the acute temporal changes in excisional murine cutaneous wound inflammation by screening of the wound-edge transcriptome. Physiol. Genom..

[13] Sugiyama S., Nakabayashi K., Baba I., Sasazuki T., Shirasawa S. (2005). Role of epiregulin in peptidoglycan-induced proinflammatory cytokine production by antigen presenting cells. Biochem. Biophys. Res. Commun..

[14] He M., Jin Q., Chen C., Liu Y., Ye X., Jiang Y., Ji F., Qian H., Gan D., Yue S. (2019). The miR-186-3p/EREG axis orchestrate tamoxifen resistance and aerobic glycolysis in breast cancer cells. Oncogene.

[15] Wang Y., Jing Y., Ding L., Zhang X., Song Y., Chen S., Zhao X., Huang X., Pu Y., Wang Z. (2019). Epiregulin reprograms cancer-associated fibroblasts and facilitates oral squamous cell carcinoma invasion via JAK2-STAT3 pathway. J. Exp. Clin. Cancer Res..

[16] Bild A., Yao G., Chang J., Wang Q., Potti A., Chasse D., Joshi M.B., Harpole D., Lancaster J., Berchuck A. (2006). Oncogenic pathway signatures in human cancers as a guide to targeted therapies. Nature.

[17] Ahmed S., Pati S., Le D., Haider K., Iqbal N. (2020). The prognostic and predictive role of 21-gene recurrence scores in hormone receptor-positive early-stage breast cancer. J. Surg. Oncol..

[18] Albanell J., Gonzalez A., Ruiz-Borrego M., Alba E., Garcia-Saenz J.A., Corominas J.M., Burgues O., Furio V., Rojo A., Palacios J. (2012). Prospective transGEICAM study of the impact of the 21-gene recurrence score assay and traditional clinicopathological factors on adjuvant clinical decision making in women with estrogen receptor-positive (ER1) node-negative breast cancer. Ann. Oncol..

[19] Goldring J.P.D. (2019). Measuring protein concentration with absorbance, Lowry, Bradford Coomassie blue, or the Smith bicinchoninic acid assay before electrophoresis. Electrophoretic Separation of Proteins.

[20] Lavorato-Rocha A.M., Anjos L.G., Cunha I.W., Vassallo J., Soares F.A., Rocha R.M. (2015). Immunohistochemical assessment of PTEN in vulvar cancer: Best practices for tissue staining, evaluation, and clinical association. Methods.

[21] Braden A.M., Stankowski R.V., Onitilo A.A. (2014). Breast cancer biomarkers: Risk assessment, diagnosis, prognosis, prediction of treatment efficacy and toxicity, and recurrence. Curr. Pharm. Des..

[22] Lee Y., Dutta A. (2009). MicroRNAs in cancer. Annu. Rev. Pathol..

[23] Wei C., Zhang R., Cai Q., Gao X., Tong F., Dong J., Hu Y. (2019). MicroRNA-330-3p promotes brain metastasis and epithelial-mesenchymal transition via GRIA3 in non-small cell lung cancer. Aging.

[24] Zhao X., Chen Q., Cao M. (2019). Abnormal expression and mechanism of miR-330-3p/BTG1 axis in hepatocellular carcinoma. Eur. Rev. Med. Pharmacol. Sci..

[25] Mesci A., Huang X., Taeb S., Jahangiri S., Kim Y., Fokas E., Bruce J., Leong H., Liu S. (2017). Targeting of CCBE1 by miR-330-3p in human breast cancer promotes metastasis. J. Br. J. Cancer.

[26] Liu B., Du R., Zhou L., Xu J., Chen S., Chen J., Yang X., Liu D., Shao Z., Zhang L. (2018). MiR-200c/141 regulates breast cancer stem cell heterogeneity via targeting HIPK1/β-Catenin axis. Theranostics.

[27] Roy S., Gonugunta V., Bandyopadhyay A., Rao M., Goodall G., Sun L., Tekmal R., Vadlamudi R. (2014). Significance of PELP1/ HDAC2/miR-200 regulatory network in EMT and metastasis of breast cancer. Oncogene.

[28] Liu Y., Li M., Yu H., Piao H. (2020). LncRNA CYTOR promotes tamoxifen resistance in breast cancer cells via sponging miR-125a-5p. Int. J. Mol. Med..

[29] Sun D., Zhong J., Wei W., Liu L., Liu J., Lin X. (2020). Long non-coding RNAs lncANGPTL-3:3 and lnc-GJA10-12:1 present as regulators of sentinel lymph node metastasis in breast cancer. Oncol. Lett..

[30] Komurasaki T., Toyoda H., Uchida D., Morimoto S. (1997). Epiregulin binds to epidermal growth factor receptor and ErbB-4 and induces tyrosine phosphorylation of epidermal growth factor receptor, ErbB-2, ErbB-3 and ErbB-4. Oncogene.

[31] Lin C., Hsieh P., Chou C., Yang C., Lee S., Tian Y., Shiue Y., Li W. (2020). High EREG expression is predictive of better outcomes in rectal cancer patients receiving neoadjuvant concurrent chemoradiotherapy. Oncology.

[32] Wang X., Li X., Lin F., Sun H., Lin Y., Wang Z., Wang X. (2021). The lnc-CTSLP8 upregulates CTSL1 as a competitive endogenous RNA and promotes ovarian cancer metastasis. J. Exp. Clin. Cancer Res..

[33] Wu X., Sui Z., Zhang H., Wang Y., Yu Z. (2020). Integrated analysis of lncRNA-mediated ceRNA network in lung adenocarcinoma. Front. Oncol..

[34] Pastushenko I., Blanpain C. (2019). EMT transition states during tumor progression and metastasis. Trends Cell Biol..

[35] Kang E., Seo J., Yoon H., Cho S. (2021). The post-translational regulation of epithelial-mesenchymal transition-Inducing transcription factors in cancer metastasis. Int. J. Mol. Sci..

[36] Zhang T., Zhou Y., You B., You Y., Yan Y., Zhang J., Pei Y., Zhang W., Chen J. (2021). miR-30a-5p inhibits epithelial-to-mesenchymal transition by targeting CDK6 in nasal polyps. Am. J. Rhinol. Allergy.

[37] Guo S., Zeng H., Huang P., Wang S., Xie C., Li S. (2018). MiR-508-3p inhibits cell invasion and epithelial-mesenchymal transition by targeting ZEB1 in triple-negative breast cancer. Eur. Rev. Med. Pharmacol. Sci..

[38] Noyan S., Ozketen A., Gurdal H., Dedeoglu B. (2021). miR-770-5p regulates EMT and invasion in TNBC cells by targeting DNMT3A. Cell. Signal..

[39] Loibl S., Poortmans P., Morrow M., Denkert C., Curigliano G. (2021). Breast cancer. Lancet.

[40] Zubair M., Wang S., Ali N. (2021). Advanced approaches to breast cancer classification and diagnosis. Front. Pharmacol..

