# Supplementary material for: NONHSAT021545/miR-330-3p/EREG: A Cooperative Axis in Breast Cancer Prognosis and Treatment
Source: J Clin Med. 2023 Mar 24;12(7):2478. doi: 10.3390/jcm12072478 (PMC10094950; doi:10.3390/jcm12072478)
Supplement: Supplementary file 1 [file jcm-12-02478-s001.zip › jcm-2192141-supplementary.pdf]

Supplementary Material

NONHSAT021545/miR-330-3p/EREG: a cooperative axis in breast cancer prognosis and treatment

Yunkun Zhang[1,2†], Chunmei Guo[3†], Siwen Yang[3†], Maroua Elkharti[1], Rui Liu[3], Ming-Zhong Sun[3*], Shuqing Liu[1*]

1 Supplementary Tables

TABLE S1 | Sequences of si-RNA for EREG and lnc021545 knockdown

Gene	si-RNA duplex oligoribonucleotide sequence
si-lnc021545-1	Forword: 5'-GCACCAAUCAACUACUUAUTT-3'<br>Reverse: 5'-AUAAGUAGUUGAUUGGUGCTT-3'
si-lnc021545-2	Forword: 5'-GCACAAACAUAGAGCCAAATT-3'<br>Reverse: 5'-UUUGGCUCUAUGUUUGUGCTT-3
si-EREG-1	Forward:5'-GCUCAAGUGUCAAUAACAATT-3'<br>Reverse: 5'-UUGUUAUUGACACUUGAGCTT-3'
si-EREG-2	Forword:5'-CCACCAACCUUUAAGCAAATT-3'<br>Reverse:5'-UUUGCUUAAAGGUUGGUGGTT-3'
si-EREG-3	Forword: 5'-GUACAGAAAUCGAAAAAGUTT-3'<br>Reverse: 5'-ACUUUUUCGAUUUCUGUACTT-3'
si-NC	Forword: 5'-UUCUCCGAACGUGUCACGUTT-3'<br>Reverse: 5'-ACGUGACACGUUCGGAGAATT-3'

TABLE S2 | Sequences of primers and oligonucleotides

Gene	Primer sequences
miR-330-3p	RT: 5'-CTCAACTGGTGTCGTGGAGTCGGCAATTCAGTGAGTCTCTGC-3'<br>Forword: 5'-ACACTCCAGCTGGGGCAAAGCACACGGCCTG-3'<br>Reverse: 5'-CTCAACTGGTGTCGTGGA-3'
U6	RT: 5'-CGCTTCACGAATTTGCGTGTCAT-3'<br>Forword: 5'-CTCGCTTCGGCAGCACA-3'<br>Reverse:5'-AACGCTTCACGAATTTGCGT-3'
lnc021545	Forword: 5'-CAGATGGTTCAAAAGTGCAAT-3'<br>Reverse: 5'-TAAAGCCAGATACTCCCAAGGAC-3'
EREG	Forword: 5'-GGACAGTGCATCTATCTGGTGGA-3'<br>Reverse: 5'-AGTGTTCACATCGGACACCAGTA-3'
β-actin	Forword: 5'-AGGCCAACCGCGAGAAG-3'<br>Reverse: 5'-ACAGCCTGGATAGCAACGTACA-3'
